# Personal protective equipment simulation training is associated with lower COVID-19 infection among healthcare workers

**DOI:** 10.31744/einstein_journal/2023AO0300

**Published:** 2023-04-18

**Authors:** Thomaz Bittencourt Couto, Paula Dias de Toledo Rodovalho Menezes, Joyce Kelly Barreto Silva, Priscilla Cerullo Hashimoto, Euma Ferreira de Sousa, Selma Tavares Valério, Etienne Larissa Duim, Simone Cristina Azevedo Silva, Lívia Almeida Dutra, Claudia Szlejf

**Affiliations:** 1 Hospital Israelita Albert Einstein São Paulo SP Brazil Hospital Israelita Albert Einstein, São Paulo, SP, Brazil.; 2 Faculdade Israelita de Ciências da Saúde Albert Einstein Hospital Israelita Albert Einstein São Paulo SP Brazil Faculdade Israelita de Ciências da Saúde Albert Einstein, Hospital Israelita Albert Einstein, São Paulo, SP, Brazil.

**Keywords:** COVID-19, Coronavirus infections, Pandemics, Education, distance, Health personnel, Simulation training, Personal protective equipment, Inservice training

## Abstract

**Objective:**

To describe the personal protective equipment training strategies during the beginning of the pandemic and to investigate the association between training and COVID-19 infection among healthcare workers.

**Methods:**

This cross-sectional study conducted between March and May 2020 included 7,142 healthcare professionals who were eligible for both online and face-to-face simulation-based training on personal protective equipment use. Simulation training attendance was assessed by reviewing the attendance list, and the COVID-19 sick leave records recovered from the institutional RT-PCR database used to grant sick leave. The association between personal protective equipment training and COVID-19 was investigated using logistic regression, adjusted for sociodemographic and occupational characteristics.

**Results:**

The mean age was 36.9 years (± 8.3), and 72.6% of participants were female. A total of 5,502 (77.0%) professionals were trained: 3,012 (54.7%) through online training, 691 (12.6%) through face-to-face training, and 1,799 (32.7%) through both strategies. During the study period, 584 (8.2%) COVID-19 cases were diagnosed among these professionals. The number of positive RT-PCR tests was 180 (11.0%) for untrained professionals, 245 (8.1%) for those trained only online, 35 (5.1%) for those trained face-to-face, and 124 (6.9%) for those trained with both strategies (p<0.001). Participants who received face-to-face training had a 0.43 lower risk of contracting COVID-19.

**Conclusion:**

Personal protective equipment training decreased the odds of COVID-19 among healthcare professionals, with face-to-face simulation-based training being most effective.

**Figure:**
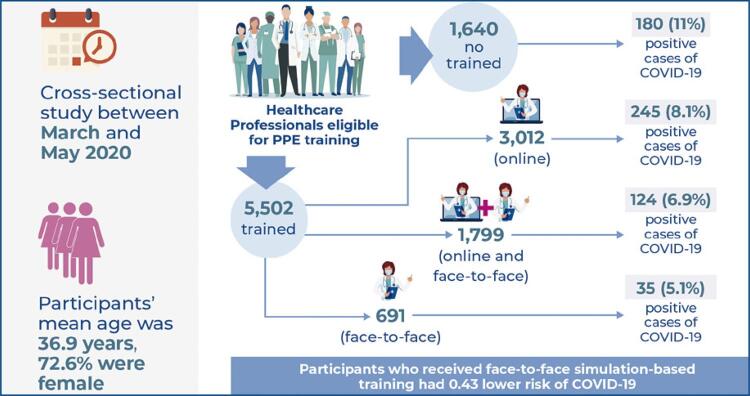

## INTRODUCTION

In January 2020, a novel coronavirus (COVID-19) was identified as being responsible for the outbreak of pneumonia and severe acute respiratory syndrome coronavirus 2 (SARS-CoV-2) in China. After two months, COVID-19 was recognized as a pandemic, with cases reported worldwide^(1)^ and a severe impact on global healthcare systems. Initially, approximately 20% of COVID-19 patients required hospitalization due to severe acute respiratory syndrome and 5% required ventilatory support, particularly those older than 65 years, with diabetes, hypertension and renal or cardiac failure.^(2)^ The COVID-19 pandemic resulted in the collapse of numerous national health care systems, as many severely ill patients required an increasing number of intensive care beds, respiratory therapy, laboratory testing and healthcare providers.^(3,4)^

First, data from China, Italy, and other smaller reports reported infection rates between 10-20% among frontline healthcare professionals.^(5,6)^ High transmissibility through droplets and aerosols, viral shedding in urine and stools, and inadequate use of personal protective equipment (PPE) are related to a higher risk of contamination.^(6)^ Preparing a response to the COVID-19 pandemic in healthcare institutions involves creating and implementing clinical protocols, adapting infrastructure, and training the workforce to safely deliver appropriate care. Educational programs that target the workforce play a central role in achieving these goals. Simulation-based education uses structured activities that represent real situations, thus allowing participants to develop their skills in a simulated environment.^(7)^ Studies demonstrated that placement and removal of PPE in simulated situations reduce the risk of contagion; moreover, it has been shown that simulation PPE training for protection against highly infective diseases is more effective at reducing donning and doffing PPE errors when compared to PPE training delivered by oral instructions or videos.^(8)^ Although current technologies cater to online education formats,^(9,10)^ training in specific skills, such as donning and doffing PPE, is likely to be more effective when delivered as face-to-face simulation-based education.^(11,12)^ Nonetheless, the social distancing measures imposed by the pandemic present an additional challenge, as the risk of contamination during face-to-face simulation-based training is unknown.

In March 2020, the Brazilian Ministry of Health declared community transmission in the country,^(2)^ and a few months later, Brazilian official pandemic numbers were at the top of worldwide statistics.^(13)^ During the initial months of the pandemic, our site, a large health system in São Paulo, Brazil, delivered PPE training to healthcare professionals using multiple educational strategies, such as face-to-face simulation and/or online training.

We hypothesized that PPE training would be associated with lower odds of COVID-19 infection among frontline healthcare workers.

## OBJECTIVE

To describe the personal protective equipment training strategies developed for healthcare professionals at the beginning of the pandemic and investigate the association between training modalities and COVID-19 infection in the healthcare workforce.

## METHODS

### Study design, setting and population

This cross-sectional study was conducted at the *Sociedade Beneficiente Israelita Brasileira Albert Einstein* (SBIBAE) in Sao Paulo, Brazil, from March to May 2020. The SBIBAE is a large Brazilian health system dedicated to healthcare, teaching, research, and philanthropy. SBIBAE provides all levels of care in the private and public sectors of São Paulo and other Brazilian cities, and its workforce totals approximately 16,000 employees. All 7,279 health care professionals working in the health system during the study period (such as physicians, nurses, nurse assistants, nurse technicians, psychologists, physical, speech, and occupational therapists) were eligible to participate in the PPE educational program. We excluded 137 healthcare professionals who were diagnosed with COVID-19 before the training sessions. Personal protective equipment training was offered face-to-face and/or online.

Training was disseminated by institutional email and through official communication platforms such as Workplace from Meta^®^ (https://work.workplace.com/). In addition, the leadership of different departments was personally notified of the availability of online and face-to-face simulation-based training to encourage employee participation.

### PPE face-to-face simulation-based training

The participants self-scheduled face-to-face training through the institution’s intranet platform. Sessions were held in the institution’s simulation center or *in situ* (work units). The sixty-minute training sessions consisted of a 15-minutes brief theoretical presentation on the COVID-19 pandemic, followed by a demonstration of the following PPE steps: hand hygiene, cap placement and removal, and instructions related to the use of N-95 respirator masks, protective goggles, shield-face, disposable waterproof coverall, and gloves. Subsequently, participants engaged in a 40-minutes task training session, in which the PPE donning and doffing sequence was repeated by each participant under the instructor’s and peer’s observation. Feedback was provided to all participants at the end of the session. Printed material for further reference and videos, including the ideal sequence of PPE placement and removal, were made available. For safety issues, training sessions were held with a limit of 20 participants per group, according to social distancing recommendations. All participants and instructors wore surgical masks and were held at a minimal distance of 1m from each other.

### PPE online training

All healthcare professionals were automatically enrolled in online training, although their participation was not universal. The self-paced online training was composed of recorded videos with ideal donning and doffing of PPE in different scenarios, as well as a theoretical explanation of COVID-19 transmissibility. A post-test comprising a multiple-choice questionnaire was also applied. The estimated time to complete training was 30 minutes.

### Identification of COVID-19 cases among participants

By the beginning of March, when community transmission was declared in the city of São Paulo, COVID-19 reverse-transcriptase polymerase chain reaction (RT-PCR) tests using nasopharyngeal swabs were performed on symptomatic employees. Additionally, all the infected employees were followed by a care coordination team from the institutional primary care network available to the workforce. The diagnosis of COVID-19 infection among study participants was based on RT-PCR results and was obtained from the occupational health database. Participants whose date of confirmatory RT-PCR preceded the date of the first face-to-face or online training session or succeeded it for up to five days, were considered as non-trained (considering a mean incubation time of five days preceding the beginning of symptoms and the time to seek testing).

### Sociodemographic and occupational characteristics

The following variables were compiled from the human resource payroll database: gender, age, education level, occupation, and work unit (emergency room, intensive care unit, wards, or other units).

### Statistical analysis

Data are presented as mean±SDs for continuous variables, and as absolute and relative frequencies for categorical variables. Participants were divided into groups according to PPE training status: PPE non-trained (PPEnt) and PPE trained (PPEt). Normally distributed continuous variables and categorical variables were compared between groups using the Student’s *t* test and χ^2^ test, respectively.

Multivariate logistic regression analysis was used to investigate the association between PPE training and COVID-19, adjusting for sociodemographic and occupational characteristics. In addition, we investigated the association between each PPE training modality (online only training, face-to-face only training, and both online and face-to-face training) and COVID-19, using the Non-trained Group as the reference and adjusting for the same set of variables. Statistical analyses were performed using Stata, version 15.1 (College Station, TX, StataCorp LLC).

### Ethics

This study was approved by the Research Ethics Committee of *Hospital Israelita Albert Einstein* (HIAE) CAAE: 32352720.2.0000.0071, # 4.161.498. The requirement for informed consent was waived because each series of data (training, employees, and hospital epidemiological) was anonymized and received confidential treatment.

## RESULTS

This study included 7,142 healthcare professionals, with a mean age of 36.9±8.3 years, and of which 72.6% were female. A total of 5,502 participants (77.0%) were included in the PPEt group, of which 691 (12.6%) were in the face-to-face only training group, 3,012 (54.7%) were in the online-only training group, and 1,799 (32.7%) were in both the online and face-to-face training groups. Most participants were nurse assistants and technicians (Table 1). There was a higher number of women, physicians, and professionals without undergraduate education, and a lower number of employees working in the emergency department and hospital wards in the PPEt group compared to the PPEnt group (Table 1). All face-to-face training sessions were evaluated by learners after completion, on a 0-10 scale to the affirmative “How would you rate your overall learning experience”, with a mean score of 9.6. No evaluation was conducted for online training.

**Table 1:** Characteristics of healthcare professionals according to personal protective equipment training

	Total	PPE training		p value

		No	Yes	
PPE training, n (%)	7,142	1,640	5,502	
Sociodemographic characteristics				
Male, n (%)	1,957 (27.4)	580 (35.4)	1,377 (25.0)	<0.001
Age (years), mean±SD	36.9±8.3	39.5±9.1	36.2±7.9	0.665
Education, n (%)				
Graduate school or above	3,332 (46.7)	558 (34.0)	2,774 (50.4)	<0.001
Occupational characteristics				
Occupation, n (%)				
Physician	1,278 (17.9)	596 (34.7)	709 (12.9)	<0.001
Nurse	1,685 (23.6)	309 (18.8)	1,376 (25.0)	
Nursing assistant	3,215 (45.0)	521 (31.8)	2,694 (49.0)	
Therapists*	486 (6.8)	133 (8.1)	353 (6.4)	
Other health professionals	478 (6.7)	108 (6.6)	370 (6.7)	
Work unit, n (%)				
Intensive care unit	91 (1.3)	56 (3.4)	35 (0.6)	<0.001
Emergency department	918 (12.9)	163 (9.9)	755 (13.7)	
Hospital wards	850 (11.9)	105 (6.4)	745 (13.6)	
Other units	5,283 (74.0)	1,316 (80.2)	3,967 (72.1)	
COVID-19 in the study period, n (%)	584 (8.2)	180 (11.0)	404 (7.3)	<0.001

* Therapists included physical therapists, occupational and speech therapists, and psychologists.

PPE: personal protective equipment; SD: standard deviation.

**Figure 1:**
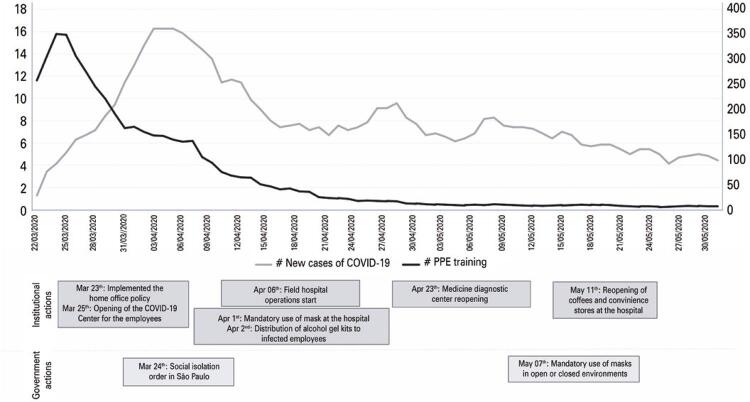
Distribution of daily new cases of COVID-19 Infection in healthcare professionals, number of trainings in personnel protective equipment use for COVID-19 delivered and institutional and governmental actions for COVID-19 prevention

**Figure 2:**
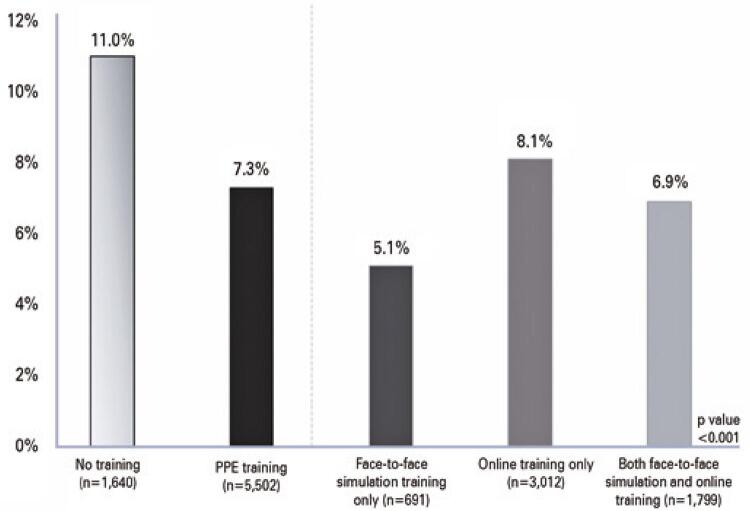
Percentual of COVID-19 infection among healthcare professionals according to personnel protective equipment training status

Figure 1 reports the daily number COVID-19 cases among the study participants and the number of trained participants per day. Between March 16th and May 31st, 584 (8.2%) participants were diagnosed with COVID-19: 404 (7.3%) in the PPEt Group and 180 (11.0%) in the PPEnt Group (p<0.001). Among the trained professionals, the number of positive tests was 245 (8.1%) for those trained online only, 35 (5.1%) for those trained face-to-face only, and 124 (6.9%) for those trained with both strategies (p<0.001), as shown in figure 2.

PPE: personal protective equipment.

Logistic regression models adjusted for age, sex, formal education, occupation, and work unit showed that healthcare workers who underwent PPE training had a 0.55-lower risk of COVID-19 than participants without training. All PPE training modalities were associated with lower odds of COVID-19 infection after adjustment (Table 2).

**Table 2:** Association of personal protective equipment training with COVID-19 infection (n=7,142)

	Crude	Adjusted*	Total (n)	COVID-19 infection, n (%)

	OR (95%CI)	OR (95%CI)		
Any PPE training	0.64 (0.53-0.77)	0.55 (0.45-0.67)	5,502	404 (7.3)
Face-to-face simulation training only	0.43 (0.30-0.63)	0.41 (0.28-0.60)	691	35 (5.1)
Online training only	0.72 (0.59-0.88)	0.64 (0.52-0.80)	3,012	245 (8.1)
Face-to-face simulation and online training	0.60 (0.47-0.76)	0.46 (0.36-0.60)	1,799	124 (6.9)
Reference: no training	1	1	1,640	180 (11.0)

* Logistic regression model adjusted for sex, age, education, occupation, and work unit.

OR: odds ratio; 95%CI: 95% confidence interval; PPE: personal protective equipment.

## DISCUSSION

In this cross-sectional study, we showed that healthcare professionals from a large Brazilian health system who underwent online and/or face-to-face simulation-based PPE training had a 0.55-lower risk of contracting COVID-19 than professionals who did not undergo PPE training. Additionally, we demonstrated that all types of PPE training decreased the odds of COVID-19 among healthcare professionals. These findings further strengthen the existing evidence on the effectiveness of educational support for healthcare workers when preparing to respond to a pandemic. Moreover, these results indicate that face-to-face simulation-based training is feasible and safe even during social distancing measures.

The lower odds of COVID-19 cases among trained participants might also be related to the simultaneous implementation of other institutional and public health measures, such as mandatory use of masks, rearrangement of the workplace, large-scale implementation of remote work for high-risk employees, administrative personnel or healthcare professionals who were not working on the frontline at the time, social distancing, and closing of non-essential businesses. However, we believe that PPE training could have contributed to the reduced odds of COVID-19 infection, reflecting a better understanding of disease transmissibility and measures of disease mitigation. While the number of cases decreased among the study participants, the incidence of the disease progressively increased in the city of São Paulo during May 2020, peaked in July 2020, and plateaued in August 2020.^(14)^ Nonetheless, as this was a cross-sectional study, it was not possible to establish a causal relationship. Moreover, although only one-third of eligible professionals were willing to participate in face-to-face training, online-only training was also associated with reduced odds of COVID-19, indicating that different strategies could effectively be used to escalate the coverage of the educational program.

Studies on frontline healthcare workers during the COVID-19 pandemic show a lack of knowledge and training on PPE use and a low competency in PPE donning and doffing.^(15,16)^ Evidence on the association between PPE training and clinical outcomes for healthcare professionals is scarce, and methods of simulated contamination may overestimate or underestimate the risk of pathogen transmission.^(17)^ We believe that the lower odds of COVID-19 infection among face-to-face simulation-based training participants could be due to the learning method itself, as studies have demonstrated that active learning provides better knowledge retention and satisfaction. Specifically, face-to-face simulation offers peer interaction and feedback, which enhances learning.^(12)^ However, the 95% confidence intervals of the odds ratios of each training method overlap, it is therefore not possible to definitively conclude that face-to-face is superior to online training. Online training is scalable, safer, and cheaper than face-to-face training and has been proven to be a reasonable educational strategy during the pandemic. Considerations on training design are important due to cost and safety, and distance training has become more attractive than face-to-face training.^(8)^

According to Kirkpatrick, training program results are evaluated on four levels:1, reaction; 2, learning; 3, behavior; and 4, results. Few simulation-based training studies have evaluated behavior and the results, the clinical impacts of training.^(18)^ Participants were satisfied with our face-to-face training, and although we did not collect level 2 outcomes, the strength of our study is its evaluation of the association between training and real life clinical outcomes. In a recent review of medical education interventions in response to the COVID-19 pandemic, among 127 studies, only seven reported level 4 outcomes, of which six reported changes in organizational practices and only one reported clinical outcomes associated with training^(19)^ (including standardization of infection control that resulted in zero cases of COVID-19 among healthcare professionals in Hong Kong).^(20)^

This study had some limitations. Its cross-sectional design precluded the establishment of a temporal relationship between training and COVID-19 diagnosis, since many participants were exposed to multiple training strategies. Further longitudinal studies should be designed to better understand the impact of PPE training on incident infections. Additionally, the study only assessed COVID-19 cases among employees tested by the institution, and a few cases were probably missed. Another relevant issue was the absence of control over PPE training prior to the pandemic and the lack of measurement of confounders related to lifestyle and behaviors outside the work environment, since transmissibility might be higher in the community.^(21)^

## CONCLUSION

In conclusion, we demonstrated that personal protective equipment training, regardless of delivery method, was associated with lower odds of COVID-19 infection among healthcare professionals in a large Brazilian health system during the first ascending months of the pandemic. Further research should be conducted to investigate the longitudinal association between personal protective equipment training and COVID-19 infection rates. Randomized clinical trials could also be designed to investigate the efficacy of such interventions, and a cost-effectiveness analysis could be further derived. In addition, prospective and comparative studies that adopt multiple training methods are needed to further elucidate the potential benefits of each intervention.
